# Acute caffeine intake increases fasciculation frequency in healthy adults: A quantitative muscle ultrasound study

**DOI:** 10.1016/j.cnp.2026.07.012

**Published:** 2026-07-21

**Authors:** David Czell, Melina Anina Brandenberger

**Affiliations:** a University of Zurich, Rämistrasse 71, 8006 Zurich, Switzerland; b Department of Neurology, GZO Hospital Wetzikon, Spitalstrasse 66, 8620 Wetzikon, ZH, Switzerland; c NeuroMedics, Loren-Allee 22, 8610 Uster, Switzerland

**Keywords:** Benign fasciculations, Caffeine, Healthy adults, Ultrasound

## Abstract

**Objective:**

To quantify the effect of acute caffeine intake on fasciculation frequency in healthy adults using muscle ultrasound and to explore differences between muscle groups and age categories.

**Methods:**

In this monocentric intraindividual pre–post study, 54 neurologically healthy adults aged 20–50 years underwent ultrasound examination of six muscles: biceps brachii, first dorsal interosseous, abductor pollicis brevis, vastus medialis, gastrocnemius caput laterale, and abductor hallucis. Fasciculations were recorded for 120 s per muscle at baseline and repeated 45 min after administration of a single oral caffeine dose of 2 mg/kg body weight. Changes in fasciculation frequency were analyzed within individuals and compared between participants aged 20–34 and 35–50 years.

**Results:**

Acute caffeine intake was associated with an increase in fasciculation frequency across most examined muscles. The largest increases were observed in the gastrocnemius and abductor hallucis, whereas changes in the vastus medialis were minimal. Upper limb muscles showed moderate increases in fasciculation activity. Age-related differences were identified: younger participants demonstrated relatively greater increases in upper limb muscles, while older participants showed more pronounced increases in lower limb muscles. Although interindividual variability was present, the overall pattern suggested enhanced motor unit excitability following caffeine intake.

**Conclusions:**

Acute caffeine intake appears to increase ultrasound-detectable fasciculation frequency in healthy adults. The effect is muscle-dependent and may vary with age.

**Significance:**

Recent caffeine consumption may influence the detection and interpretation of fasciculations during neuromuscular assessment. Consideration of caffeine intake before muscle ultrasound or electromyographic examinations may improve diagnostic accuracy and reduce misinterpretation of benign fasciculation activity. (218 words).

## Highlights

• Acute caffeine intake increases fasciculation frequency in healthy adults.
• Muscle ultrasound detects caffeine-related increases in fasciculation activity.
• Recent caffeine intake may influence clinical interpretation of fasciculations.

## 1 Introduction

Fasciculations are spontaneous, involuntary discharges of single motor units that manifest clinically as brief, visible muscle twitches. They are a common phenomenon in neuromuscular medicine and occur in both physiological and pathological contexts (Desai and Swash, 1997; Mitsikostas et al., 1998). In pathological conditions involving the lower motor neuron, such as amyotrophic lateral sclerosis (ALS), fasciculations are often associated with progressive muscle weakness, atrophy, and hyperreflexia (De Carvalho et al., 2017; Mills, 2010). However, fasciculations are also frequently observed in neurologically healthy individuals, where they are typically considered benign (Walter, 2015; Mitsikostas et al., 1998).

This overlap between benign and pathological fasciculations represents a significant diagnostic challenge. Patients often present with isolated fasciculations and considerable anxiety, particularly due to the association of fasciculations with ALS. Previous studies have highlighted that, in the absence of additional neurological deficits, fasciculations are most commonly benign (Desai and Swash, 1997; Simon and Kiernan, 2013). Nevertheless, early stages of motor neuron disease may present with fasciculations alone, complicating clinical interpretation and necessitating careful diagnostic assessment (De Carvalho et al., 2017).

The pathophysiology of fasciculations is complex and not yet fully understood. Proposed mechanisms include spontaneous depolarization of anterior horn cells, distal axonal hyperexcitability, and instability of the motor unit membrane (Mills, 2010; Amato and Russell, 2016). In pathological conditions, increased excitability of the motor neuron has been linked to both central and peripheral mechanisms, including glutamatergic excitotoxicity and impaired inhibitory signaling (De Carvalho et al., 2017). However, similar mechanisms may also be active in physiological conditions under certain stimuli.

Electromyography (EMG) remains the gold standard for detecting fasciculation potentials and allows detailed characterization of motor unit firing patterns. However, EMG is invasive, limited to relatively small sampling areas, and may fail to detect intermittent fasciculations during short recording periods (Reimers et al., 1996; Bokuda et al., 2020).

Muscle ultrasound (MUS) has emerged as a valuable complementary technique for detecting fasciculations. It enables non-invasive, real-time visualization of muscle activity across larger regions and over extended observation times (Reimers et al., 1996; Duarte et al., 2020). Fasciculations appear on ultrasound as brief, localized muscle movements lasting approximately 0.2–1.0 s (Noto et al., 2017). Several studies have demonstrated that MUS may have equal or greater sensitivity than EMG for detecting fasciculations, particularly when multiple muscles and longer observation periods are examined (Duarte et al., 2020; Noto et al., 2017).

The occurrence and distribution of fasciculations are influenced by a variety of physiological and environmental factors. Exercise, fatigue, stress, and sleep deprivation have all been associated with increased fasciculation frequency (Fermont et al., 2010; Walter, 2015). In healthy individuals, fasciculations are often more prevalent in distal muscles such as the calf and intrinsic foot muscles, possibly reflecting differences in motor unit organization, axonal length, or functional demand (Fermont et al., 2010; Czell et al., 2016).

Among modifiable external factors, caffeine is of particular relevance due to its widespread consumption. Caffeine is a methylxanthine that acts predominantly as a non-selective antagonist of adenosine receptors, particularly A1 and A2A subtypes (Ribeiro and Sebastião, 2010). Adenosine normally exerts inhibitory effects on neuronal activity; thus, its blockade results in increased neuronal firing and enhanced release of excitatory neurotransmitters such as glutamate, dopamine, and noradrenaline (Cappelletti et al., 2015; Ribeiro and Sebastião, 2010).

At the level of the motor system, caffeine has been shown to increase motor unit excitability. Experimental studies have demonstrated that caffeine administration enhances motor unit firing rates and increases the occurrence of sustained or spontaneous activity (Walsh et al., 2008; Kalmar and Cafarelli, 2004). These effects provide a plausible mechanistic explanation for the clinical observation that caffeine may exacerbate fasciculations.

Indeed, caffeine has been identified as a common provoking factor in benign fasciculation syndrome and is also recognized as a contributor to neuromuscular hyperexcitability in conditions of excessive intake (American Psychiatric Association, 2013; Walter, 2015). However, most available evidence is based on subjective symptom reporting or indirect electrophysiological measurements rather than direct visualization techniques.

Furthermore, the effect of caffeine on the distribution of fasciculations across different muscle groups has not been systematically explored. It remains unclear whether certain muscles, particularly distal versus proximal muscles, exhibit differential susceptibility to caffeine-induced changes in excitability. Similarly, potential age-related differences in fasciculation response remain poorly understood, although age-related changes in motor unit structure and function may play a role (Carmeli et al., 2003).

Given these limitations in the current literature, there is a need for quantitative and standardized assessment of the acute effects of caffeine on fasciculation activity. Muscle ultrasound provides an ideal method for such investigation, allowing direct and non-invasive visualization of fasciculations across multiple muscle groups.

The primary aim of this study was therefore to quantify the effect of acute caffeine intake on fasciculation frequency in healthy adults using muscle ultrasound. Secondary aims were to assess differences between muscle groups, particularly distal versus proximal muscles and upper versus lower extremities, and to explore potential age-related differences in fasciculation response.

By providing quantitative ultrasound-based data on caffeine-induced changes in fasciculation activity, this study aims to improve the understanding of physiological modulators of motor unit excitability and to support more accurate clinical interpretation of fasciculations in neuromuscular practice.

## 2 Methods

### 2.1 Study design and setting

This study was designed as a **monocentric, intraindividual pre–post study** to assess the acute effect of caffeine on fasciculation frequency. All examinations were conducted at a single center under standardized conditions.

The intraindividual design was chosen to minimize interindividual variability, as each participant served as their own control. This approach allows for more precise detection of changes in fasciculation frequency attributable to caffeine intake.

### 2.2 Participants

A total of **54 healthy volunteers** aged between **20 and 50 years** were included in the study. Participants were stratified into two age groups:

• younger group: 20–34 years
• older group: 35–50 years

Recruitment was performed through local advertisement and professional networks.

All participants underwent a **standardized neurological examination** prior to inclusion. This included assessment of:

• muscle strength
• deep tendon reflexes
• sensory function
• coordination

Only individuals without clinical evidence of neuromuscular disease were included.

### 2.3 Exclusion criteria

Participants were excluded if any of the following criteria were present:

• history of neurological disease
• symptoms suggestive of neuromuscular disorder
• abnormal findings on neurological examination
• use of medications known to affect central or peripheral nervous system excitability (e.g., stimulants)
• visible muscle atrophy or weakness

This rigorous screening ensured that only neurologically healthy individuals were included.

### 2.4 Baseline characteristics and potential confounders

Baseline caffeine consumption and physical activity levels were **not systematically recorded**, which represents a limitation of the study. Both factors may influence motor unit excitability and could therefore contribute to interindividual variability in fasciculation frequency.

### 2.5 Ultrasound protocol

#### 2.5.1 Equipment and settings

All ultrasound examinations were performed using a **high-resolution linear transducer** (5–15 MHz).

To ensure comparability across measurements, ultrasound parameters were kept constant:

• gain
• depth
• focus
• frame rate

Standardization of acquisition parameters is essential, as variations in settings may influence the detection of subtle muscle movements such as fasciculations.

### 2.6 Muscle selection

Six muscles were examined on the **right side of the body**:

**Upper limb muscles:**

• biceps brachii
• first dorsal interosseous
• abductor pollicis brevis

**Lower limb muscles:**

• vastus medialis
• gastrocnemius (caput laterale)
• abductor hallucis

This selection allowed the assessment of:

• proximal vs distal muscles
• upper vs lower limb distribution

### 2.7 Measurement procedure

Participants were examined in a **relaxed supine position** and instructed to avoid voluntary muscle contraction throughout the recording period.

**Fig. 1:**
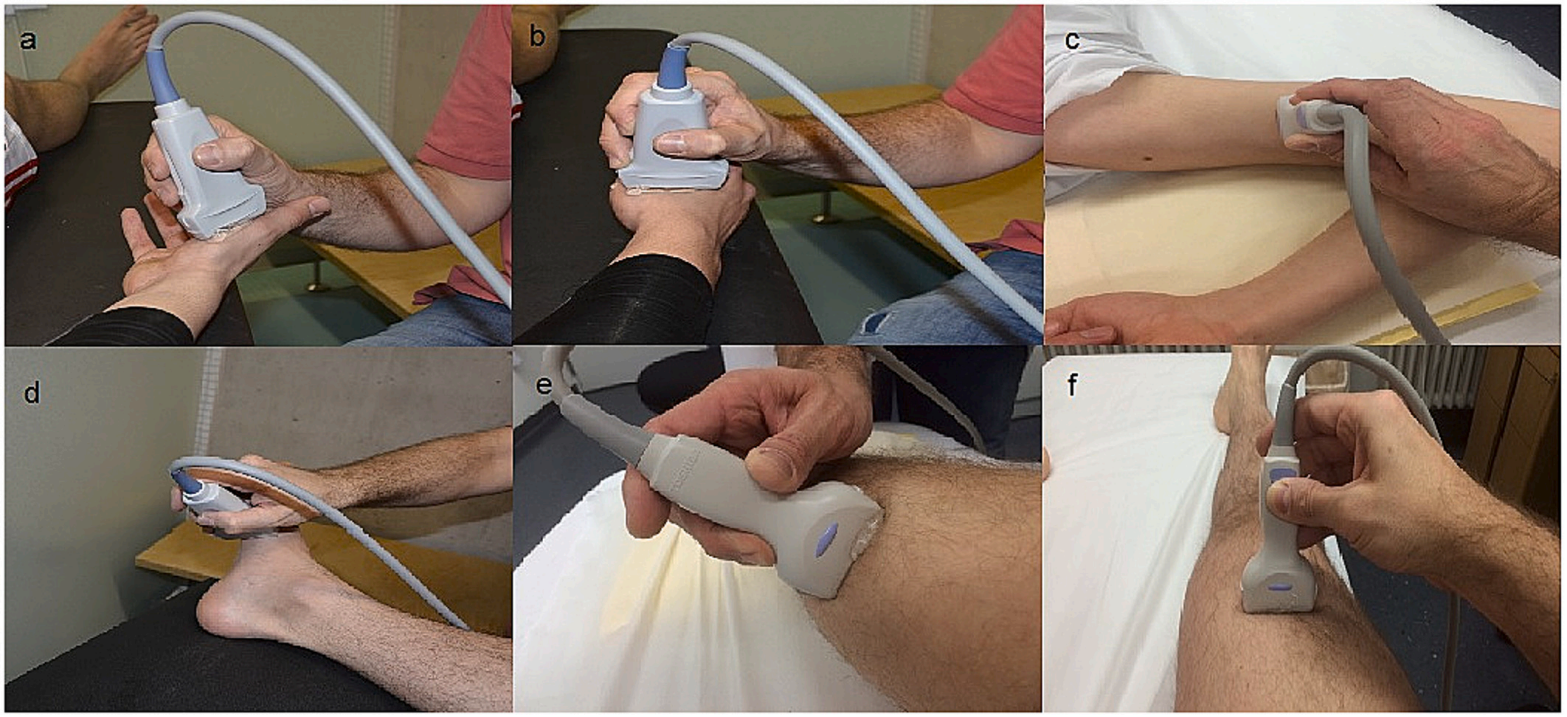
Ultrasound position of the six muscles. a) Abductor pollicis brevis; b) First dorsal interosseus; c) Bizeps brachii; d) Abductor hallucis; e) Gastrocnemius; f) Vastus medialis.

The ultrasound probe was placed at **predefined anatomical landmarks**, corresponding to the **maximal muscle belly**, to ensure reproducibility (Fig. 1).

Each muscle was observed for **120 s** with continuous video recording.

### 2.8 Definition of fasciculations

Fasciculations were defined as:

**spontaneous, focal muscle contractions lasting approximately 0.2–1.0 s.**

They appeared as localized, transient movements of muscle fibers within the ultrasound image.

Special care was taken to distinguish fasciculations from:

• repetitive discharges of the same motor unit (pseudotremor)
• movement artefacts

Only discrete, non-rhythmic contractions were counted as fasciculations.

### 2.9 Intervention (caffeine administration)

Participants were instructed to **abstain from caffeine for at least 12 h** prior to baseline assessment to standardize starting conditions.

Each participant received a **single oral dose of caffeine (2 mg/kg body weight)**.

Follow-up ultrasound examinations were performed **45 min after caffeine intake**, corresponding to expected peak plasma concentrations.

This timing was chosen based on established pharmacokinetic data for caffeine absorption.

### 2.10 Outcome measures

The primary outcome measure was:

• change in fasciculation frequency (post – pre)

This was assessed:

• per muscle
• per participant
• and across muscle groups

Secondary outcomes included:

• differences between **muscle groups** (upper vs lower limb, proximal vs distal)
• differences between **age groups**

Fasciculation frequency was quantified as the **number of fasciculations observed during the 120-s recording period** for each muscle.

### 2.11 Statistical analysis

Statistical analyses focused on intraindividual comparisons of fasciculation frequency before and after caffeine intake.

Continuous variables were summarized using means and standard deviations (SD) for approximately normally distributed data and medians with interquartile ranges (IQR) where appropriate. Normality of the distribution of paired differences (post–pre) was assessed using visual inspection of histograms and the Shapiro–Wilk test.

For pre–post comparisons, a paired *t*-test was used when the distribution of paired differences did not significantly deviate from normality. If the normality assumption was violated, the Wilcoxon signed-rank test was applied. Analyses were performed for each muscle separately and for overall fasciculation counts.

Comparisons between the two age groups (20–34 years and 35–50 years) were based on the change in fasciculation frequency (Δ = post–pre). Depending on data distribution, either an independent-samples *t*-test or the Mann–Whitney *U* test was used.

To account for the moderate sample size and potential deviations from normality, bootstrap resampling with 2000 iterations was performed. Bootstrap procedures were used primarily to estimate 95% confidence intervals for mean changes and effect estimates, thereby assessing the robustness of the observed findings.

Effect sizes were calculated and reported as Cohen's d for parametric analyses and rank-biserial correlations (r) for non-parametric analyses where appropriate.

All tests were two-sided, and a *p*-value <0.05 was considered statistically significant.

No formal a priori sample size calculation was performed because of the exploratory nature of the study. Consequently, the findings should be considered hypothesis-generating and interpreted with appropriate caution. Future studies with larger cohorts and predefined power calculations are required to confirm the observed effects.

### 2.12 Ethics

All participants provided **written informed consent** prior to participation.

The study protocol was reviewed and approved by the responsible regional ethics committee and conducted in accordance with the principles of the Declaration of Helsinki.

## 3 Results

### 3.1 Participant characteristics

A total of 54 healthy volunteers were included in the study. The cohort comprised 26 participants aged 20–34 years (younger group) and 28 participants aged 35–50 years (older group). Both groups were comparable with regard to sex distribution and baseline clinical examination findings. All participants completed the study protocol without adverse events.

### 3.2 Baseline fasciculation activity

At baseline, fasciculations were detected only infrequently across the examined muscles. When present, they were most commonly observed in distal lower limb muscles, particularly the **gastrocnemius caput laterale** and the **abductor hallucis** (Table 1).

**Table 1:** Descriptive statistics of the 54 subjects showing fasciculations in the lower leg muscles (M. gastrocnemius caput laterale, M. abductor hallucis).

N	Mean	SD	Median	Min	Max	Range	Skew	Kurtosis	SE
54	+10.72	5.81	10.5	−1	29	30	0.74	0.94	0.79

N = number of subjects; SD = standard deviation; SE = standard error.

Upper limb muscles—including the **biceps brachii**, **first dorsal interosseous**, and **abductor pollicis brevis**—showed minimal baseline activity, with the majority of participants demonstrating no detectable fasciculations during the 120-s observation period (Table 2).

**Table 2:** Descriptive statistics of the 54 subjects showing fasciculations in the arm muscles (M. abductor pollicis, M. interosseus dorsalis, M. biceps brachii).

N	Mean	SD	Median	Min	Max	Range	Skew	Kurtosis	SE
54	+3.17	3.45	3	0	11	11	0.72	−0.68	0.47

N = number of subjects; SD = standard deviation; SE = standard error.

The **vastus medialis** showed almost no baseline fasciculations, with only occasional low-frequency activity in a small number of individuals. Overall, baseline findings were consistent with previously described distributions of benign fasciculations in healthy populations, with a predominance in distal musculature (Table 3).

**Table 3:** Descriptive statistics of the 54 subjects showing fasciculations in the upper leg muscle (M. vastus medialis).

N	Mean	SD	Median	Min	Max	Range	Skew	Kurtosis	SE
54	0.5	1.11	0	0	5	5	2.35	5.02	0.15

N = number of subjects; SD = standard deviation; SE = standard error.

### 3.3 Effect of caffeine on fasciculation frequency

#### 3.3.1 Global analysis

Following caffeine administration, a **consistent and measurable increase in fasciculation frequency** was observed across the study population. This increase was evident in the majority of participants and affected nearly all examined muscle groups.

The overall pattern demonstrated a **positive change in fasciculation counts (post–pre)**, indicating that caffeine intake was associated with increased spontaneous motor unit activity. This effect was robust across individuals, although interindividual variability in magnitude was noted.

### 3.4 Muscle-specific effects

#### 3.4.1 Lower limb muscles

**Fig. 2:**
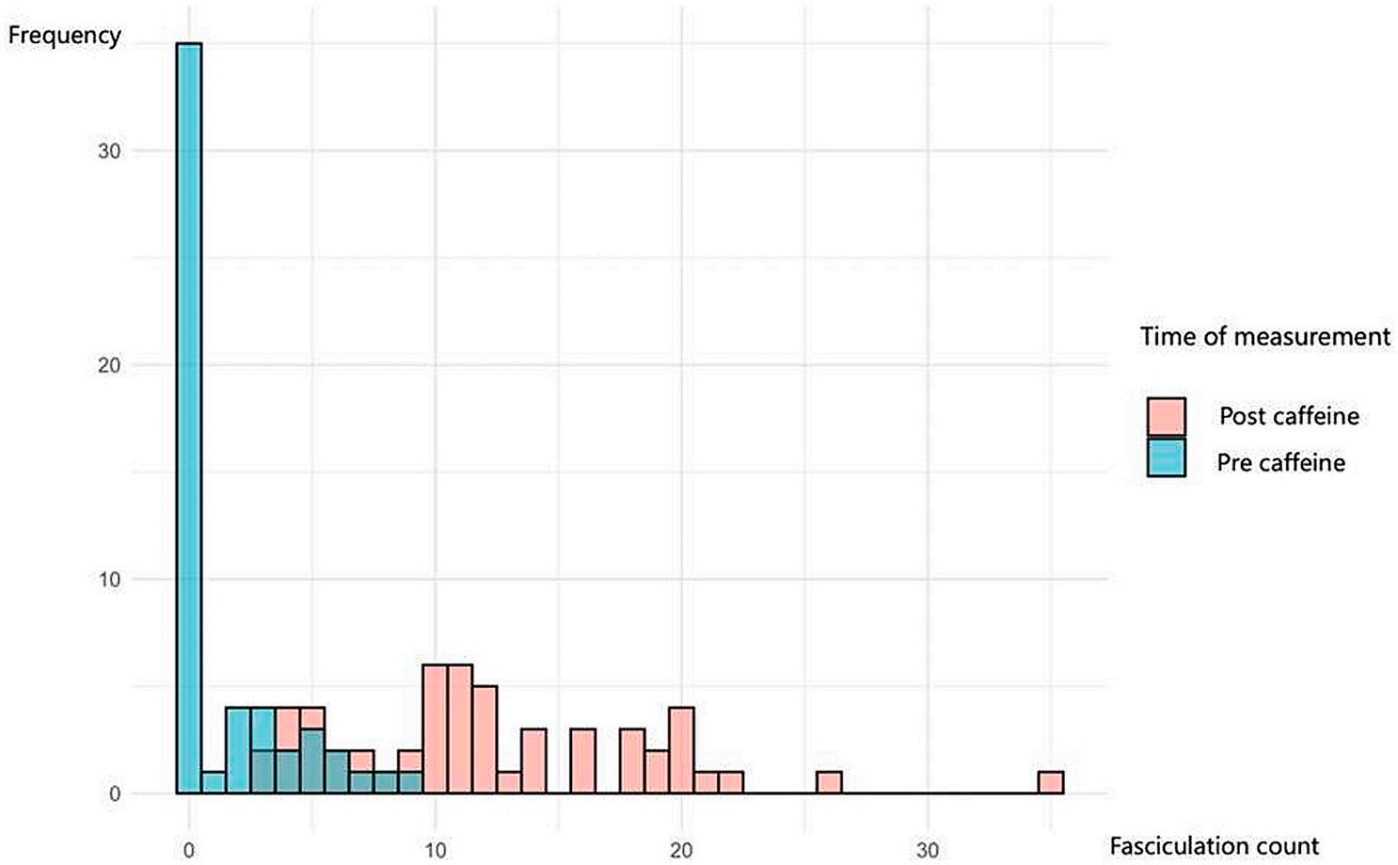
Absolute number and frequency of 54 subjects showing fasciculations in the lower leg muscles measured.

The most pronounced effects of caffeine were observed in the distal lower limb muscles (Fig. 2).

The **gastrocnemius caput laterale** showed the largest increase in fasciculation frequency, with many participants exhibiting a clear transition from absent or minimal baseline activity to frequent fasciculations after caffeine intake. In several cases, fasciculations became continuously detectable throughout the observation period.

Similarly, the **abductor hallucis** demonstrated a marked increase in activity. After caffeine intake, fasciculations were detectable in nearly all participants in this muscle, even in those without baseline activity.

**Fig. 3:**
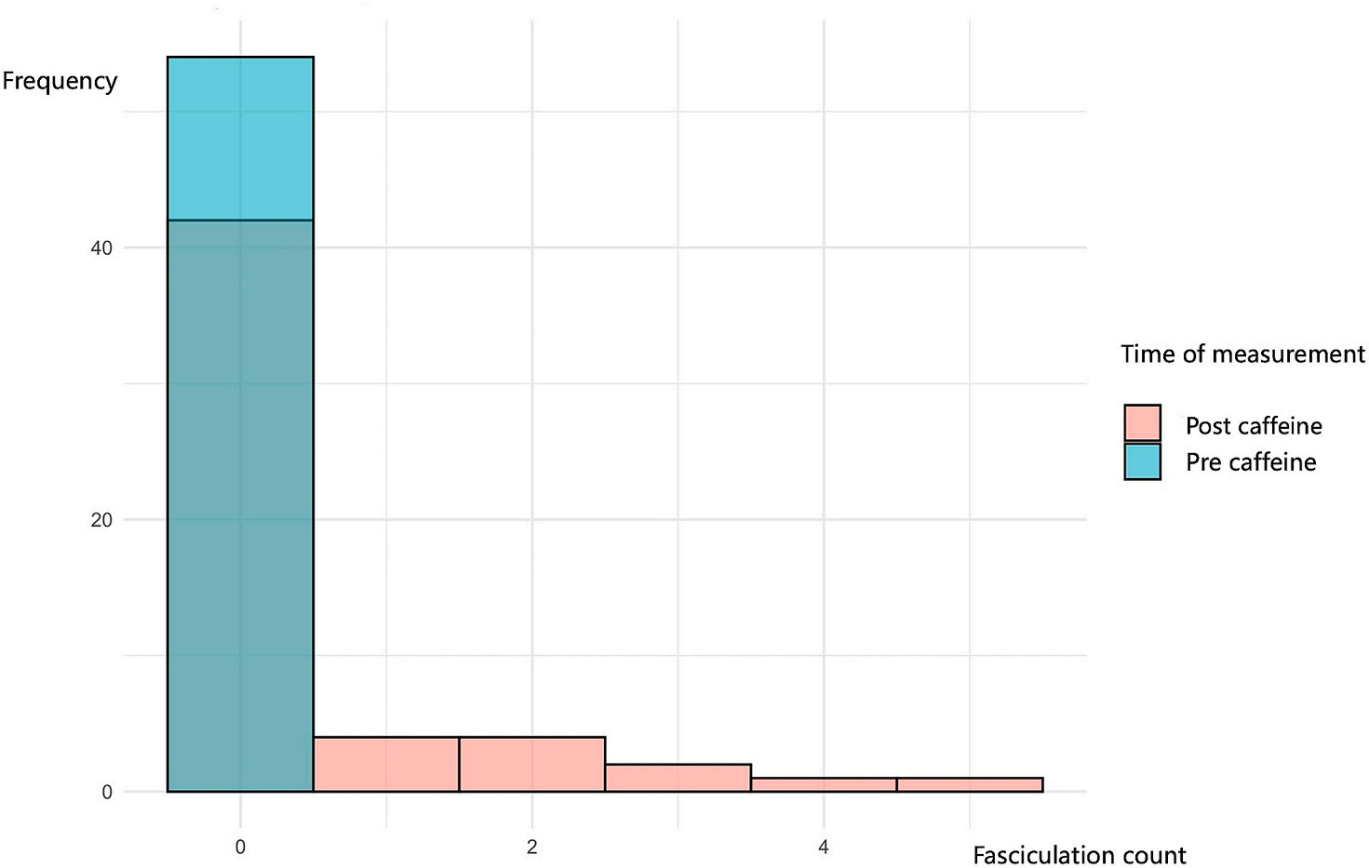
Absolute number and frequency of 54 subjects showing fasciculations in the vastus medialis muscle measured.

In contrast, the **vastus medialis** showed only minimal changes (Fig. 3). Although a slight increase in fasciculation frequency was observed in some individuals, this muscle remained largely unaffected compared to distal lower limb muscles.

#### 3.4.2 Upper limb muscles

**Fig. 4:**
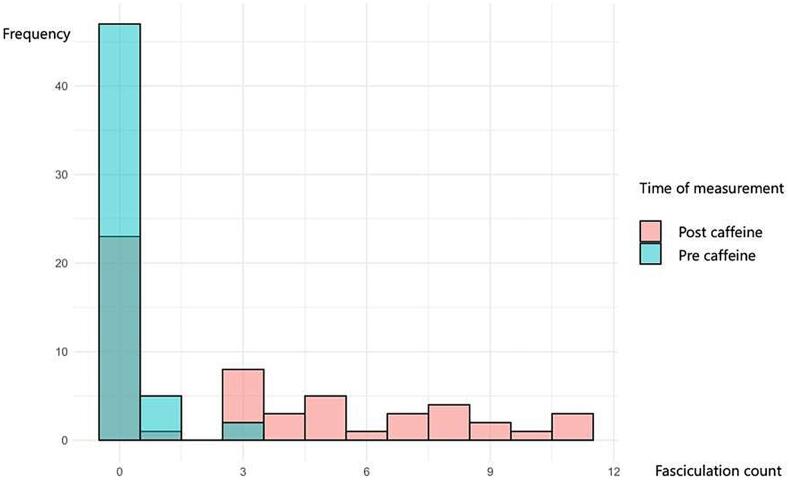
Absolute number and frequency of 54 subjects showing fasciculations in the arm muscles measured.

Upper limb muscles exhibited a **moderate but consistent increase** in fasciculation frequency following caffeine intake (Fig. 4).

The **biceps brachii** showed an increase in the occurrence of fasciculations, although the magnitude of change was smaller compared with distal lower limb muscles. Fasciculations were typically sporadic rather than sustained.

The **first dorsal interosseous** and **abductor pollicis brevis** demonstrated similar patterns, with increased detectability of fasciculations after caffeine intake. These muscles showed improved visibility of fasciculations post-intervention, but overall activity levels remained lower than those observed in the gastrocnemius and abductor hallucis.

These findings indicate that while caffeine increases excitability across muscle groups, the magnitude of response differs substantially between regions.

### 3.5 Comparison between muscle groups

When comparing muscle groups, a clear gradient of responsiveness to caffeine was observed (Table 4):

• **Highest increase**: distal lower limb muscles (gastrocnemius, abductor hallucis)
• **Intermediate increase**: upper limb muscles
• **Minimal increase**: proximal lower limb muscle (vastus medialis)

**Table 4:** Assessment of the caffeine effect by muscle group in all 54 test subjects.

Muscle group	M (SD)	95% Confidence interval	Caffeine effect
Arms	3.17 (3.45)	[2.26; 4.15]	Positive (+)
Lower Legs	10.72 (5.81)	[9.22; 12.39]	Positive (+)
M. vastus medialis	0.5 (1.11)	[0.22; 0.81]	Positive (+)

This distribution suggests that distal muscles may be more sensitive to changes in motor unit excitability induced by caffeine.

### 3.6 Age-related differences

Stratification by age group revealed distinct patterns of fasciculation response (Table 5).

**Table 5:** Assessment of the caffeine effect by muscle group according to age in all 54 test subjects.

Muscle group	20–34 y: M (SD)	35–50 y: M (SD)	95% Confidence interval of difference	Caffeine effect
Arms	4.62 (3.21)	1.82 (3.14)	[1.05; 4.44]	Positive (+)
Lower legs	8.65 (5.77)	12.64 (5.24)	[−7.01; −0.97]	Positive (+)
M. vastus medialis	0.5 (1.07)	0.5 (1.17)	[−0.56; 0.62]	Positive (+)

In the **younger cohort (20–34 years)**, the relative increase in fasciculation frequency was more pronounced in the **upper limb muscles**. These participants showed a greater post-caffeine increase in muscles such as the first dorsal interosseous and abductor pollicis brevis compared to the older group.

In contrast, the **older cohort (35–50 years)** demonstrated a relatively greater increase in **lower limb muscles**, particularly in the gastrocnemius caput laterale and abductor hallucis.

No meaningful age-related differences were observed for the vastus medialis, consistent with the generally low responsiveness of this muscle.

These findings indicate that age may influence the regional distribution of caffeine-induced changes in motor unit excitability.

### 3.7 Interindividual variability

Despite the overall consistent trend, some variability in the magnitude of response to caffeine was observed between participants.

While most individuals showed a clear increase in fasciculation frequency after caffeine intake, a smaller subset demonstrated only minimal changes. Conversely, a few participants exhibited pronounced increases across multiple muscles.

This variability may be related to unmeasured factors such as habitual caffeine consumption, individual sensitivity, or baseline neuromuscular excitability.

### 3.8 Summary of findings

In summary, acute caffeine intake resulted in:

• A global increase in fasciculation frequency in healthy individuals
• A muscle-dependent effect, with strongest increases in distal lower limb muscles
• Moderate effects in upper limb muscles
• Minimal changes in the vastus medialis
• Age-dependent differences in distribution of fasciculation increase

## 4 Discussion

The present study demonstrates that acute caffeine intake is associated with a measurable and consistent increase in fasciculation frequency in healthy individuals, as detected by muscle ultrasound. These findings align with existing knowledge regarding the effects of caffeine on neuronal and motor unit excitability and provide quantitative confirmation of this relationship using a non-invasive imaging technique.

### 4.1 Interpretation and relation to existing literature

Caffeine is a well-established central nervous system stimulant that exerts its primary effects through antagonism of adenosine receptors, particularly A1 and A2A subtypes (Ribeiro and Sebastião, 2010; Cappelletti et al., 2015). Adenosine normally acts as an inhibitory neuromodulator, reducing neuronal firing and limiting synaptic transmission. By blocking this inhibitory tone, caffeine enhances neuronal activity and increases the release of excitatory neurotransmitters, including glutamate and catecholamines (Ribeiro and Sebastião, 2010).

At the level of the motor system, this increased excitability can facilitate spontaneous motor unit discharges. Experimental studies have demonstrated that caffeine intake increases motor unit firing rates and promotes sustained or self-reinitiating activity in voluntary and involuntary motor units (Kalmar and Cafarelli, 2004; Walsh et al., 2008). These findings provide a mechanistic basis for the observed increase in fasciculation frequency following caffeine intake in the present study.

Previous clinical observations have also suggested that caffeine may exacerbate fasciculations. In benign fasciculation syndrome, caffeine is commonly reported as a triggering or aggravating factor, together with stress, fatigue, and physical exertion (Walter, 2015). Furthermore, caffeine intoxication has been associated with symptoms of neuromuscular hyperexcitability, including tremor and muscle twitching (American Psychiatric Association, 2013). However, most of these observations have relied on subjective reporting or indirect measurements, rather than direct visualization techniques.

The present study extends existing knowledge by providing **quantitative ultrasound-based evidence** that caffeine intake leads to a measurable increase in fasciculation frequency across multiple muscle groups. Importantly, these findings should be interpreted as confirmatory rather than novel, given the substantial body of physiological and clinical evidence supporting the excitatory effects of caffeine on motor neuron activity.

### 4.2 Muscle-specific differences in fasciculation response

A notable finding of this study is the marked variation in response between different muscle groups. Distal lower limb muscles, particularly the gastrocnemius and abductor hallucis, demonstrated the most pronounced increase in fasciculation frequency following caffeine intake.

This pattern is consistent with previous studies reporting higher baseline fasciculation activity in distal musculature (Fermont et al., 2010; Czell et al., 2016). Several factors may contribute to this observation. Distal muscles are characterized by longer motor axons and may therefore be more susceptible to alterations in axonal excitability. In addition, differences in motor unit density, fiber type composition, and functional demand may influence baseline excitability and responsiveness to external stimuli.

By contrast, the vastus medialis showed minimal changes in fasciculation frequency. This finding suggests that proximal muscles may be less sensitive to acute changes in excitability or may require stronger stimuli to exhibit measurable alterations. Similar muscle-specific differences have been described in ultrasound studies of fasciculations in both healthy individuals and patients with neuromuscular disorders (Noto et al., 2017).

Upper limb muscles demonstrated an intermediate response, with a consistent but less pronounced increase in fasciculation frequency compared to distal lower limb muscles. This gradient of responsiveness—distal > upper limb > proximal—supports the notion that anatomical and physiological properties of individual muscles play a key role in determining susceptibility to excitability changes.

### 4.3 Age-related differences

The present study also identified differences in the distribution of caffeine-induced fasciculation changes between age groups. Younger participants demonstrated relatively greater increases in upper limb muscles, whereas older participants showed a stronger response in lower limb muscles.

The underlying mechanisms of these differences remain unclear. Age-related changes in motor unit structure and function may play a role. It is well established that aging is associated with motor unit remodeling, including loss of motor neurons and compensatory reinnervation, leading to larger but fewer motor units (Carmeli et al., 2003). These structural changes may influence baseline excitability and responsiveness to external stimuli such as caffeine.

In addition, differences in habitual muscle use patterns between age groups may contribute to the observed distribution. Physical activity, occupational factors, and lifestyle differences may all influence regional neuromuscular excitability. However, as baseline caffeine intake and physical activity were not systematically recorded in this study, these interpretations remain speculative and should be addressed in future research.

### 4.4 Clinical implications

The findings of this study have direct implications for clinical practice. Fasciculations are frequently assessed in patients undergoing evaluation for neuromuscular disorders, particularly motor neuron disease and benign fasciculation syndrome. The presence, frequency, and distribution of fasciculations may influence diagnostic decision-making.

The observation that caffeine intake significantly increases fasciculation frequency suggests that **recent caffeine consumption may act as a confounding factor** in neuromuscular examinations. Patients often consume caffeine shortly before clinical assessment, for example in the form of coffee prior to outpatient visits. This may lead to an increased likelihood of detecting fasciculations, particularly when using sensitive methods such as muscle ultrasound.

Failure to consider this effect may contribute to **overinterpretation of benign findings** or increased diagnostic uncertainty. In particular, increased fasciculation activity in the absence of other clinical signs could be misinterpreted as indicative of pathology.

From a practical standpoint, it may therefore be advisable to recommend abstinence from caffeine for at least 12 h prior to neuromuscular evaluation when fasciculations are a primary diagnostic concern. However, this recommendation is based on pharmacokinetic considerations and the study protocol rather than direct evidence regarding the optimal duration of abstinence and should therefore be considered preliminary.

The duration of caffeine abstinence required before neuromuscular assessment remains uncertain and was not specifically addressed in the present study. Participants were instructed to abstain from caffeine for at least 12 h prior to baseline examination, which was considered sufficient to minimize acute stimulant effects. Given the pharmacokinetics of caffeine, with a plasma half-life typically ranging from approximately 3 to 7 h in healthy adults, a period of at least 12 h without caffeine appears reasonable for routine clinical examinations. However, residual effects may vary depending on individual metabolism, habitual caffeine consumption, smoking status, medication use, and hepatic function. Therefore, future studies should investigate the temporal relationship between caffeine intake and fasciculation frequency to determine the optimal abstinence period before electromyographic or ultrasound-based neuromuscular evaluations.

### 4.5 Methodological considerations

The use of muscle ultrasound as the primary detection method represents a strength of this study. MUS allows continuous observation of large muscle areas over extended periods, thereby increasing sensitivity for detecting intermittent fasciculations (Duarte et al., 2020; Noto et al., 2017).

However, certain methodological aspects should be considered. The detection of fasciculations depends on technical factors such as probe positioning, image quality, and examiner experience. Standardization of acquisition parameters is therefore essential to ensure comparability. In the present study, a single experienced examiner and standardized settings were used to minimize variability.

Furthermore, the observation period of 120 s per muscle was chosen based on previous studies suggesting that this duration provides a reasonable balance between sensitivity and feasibility (Noto et al., 2017). Nevertheless, longer observation periods may further increase detection rates.

### 4.6 Limitations

Several limitations of this study should be acknowledged.

First, baseline caffeine consumption was not systematically recorded. Individual tolerance to caffeine may influence the magnitude of response, and habitual consumption could attenuate or modify the observed effects.

Second, physical activity levels and recent exercise were not assessed. Exercise is a known modulator of fasciculation activity and may have influenced baseline measurements (Fermont et al., 2010).

Third, the study did not include a placebo control group. While the intraindividual design reduces variability, it does not completely exclude potential confounding factors such as circadian variation or anticipation effects.

Fourth, the relatively small sample size limits the statistical power of subgroup analyses, particularly regarding age-related differences. Because no formal a priori power calculation was performed, the study was designed as an exploratory investigation. The observed effects should therefore be interpreted as hypothesis-generating and require confirmation in larger, adequately powered studies.Finally, the study evaluated fasciculation frequency at a single time point (45 min) after caffeine intake and therefore does not allow conclusions regarding the duration of the observed effect or the optimal period of caffeine abstinence before neuromuscular assessment.

## 5 Conclusion

In conclusion, this exploratory study suggests that acute caffeine intake is associated with an increase in fasciculation frequency in healthy adults as detected by muscle ultrasound. While the findings are consistent with the known excitatory effects of caffeine on motor unit activity, confirmation in larger, prospectively powered studies is warranted.The observed muscle-specific and age-related differences suggest that the response to caffeine is not uniform and may depend on anatomical and physiological factors. Importantly, these findings have direct implications for clinical practice, indicating that recent caffeine intake should be considered when interpreting fasciculations in neuromuscular assessment.

Future studies should aim to further characterize the relationship between caffeine dose, habitual consumption, and fasciculation activity, as well as to explore these effects in patient populations.

## Funding sources

This research did not receive any specific grant from funding agencies in the public, commercial, or not-for-profit sectors.

## Declaration of competing interest

The authors declare that they have no known competing financial interests or personal relationships that could have appeared to influence the work reported in this paper.
